# Enhancing medulloblastoma classification with nanopore sequencing of FFPE samples

**DOI:** 10.1093/noajnl/vdaf180

**Published:** 2025-08-11

**Authors:** Marica Ficorilli, Matilde Oriani, Chiara Dossena, Federica Morelli, Marta Lucchetta, Veronica Biassoni, Elisabetta Schiavello, Olga Nigro, Maura Massimino, Loris De Cecco

**Affiliations:** Integrated Biology of Rare Tumors, Department of Experimental Oncology, Fondazione IRCCS Istituto Nazionale dei Tumori, Milan, Italy; Integrated Biology of Rare Tumors, Department of Experimental Oncology, Fondazione IRCCS Istituto Nazionale dei Tumori, Milan, Italy; Integrated Biology of Rare Tumors, Department of Experimental Oncology, Fondazione IRCCS Istituto Nazionale dei Tumori, Milan, Italy; Integrated Biology of Rare Tumors, Department of Experimental Oncology, Fondazione IRCCS Istituto Nazionale dei Tumori, Milan, Italy; Integrated Biology of Rare Tumors, Department of Experimental Oncology, Fondazione IRCCS Istituto Nazionale dei Tumori, Milan, Italy; Pediatric Oncology Unit, Fondazione IRCCS Istituto Nazionale dei Tumori, Milan, Italy; Pediatric Oncology Unit, Fondazione IRCCS Istituto Nazionale dei Tumori, Milan, Italy; Pediatric Oncology Unit, Fondazione IRCCS Istituto Nazionale dei Tumori, Milan, Italy; Pediatric Oncology Unit, Fondazione IRCCS Istituto Nazionale dei Tumori, Milan, Italy; Integrated Biology of Rare Tumors, Department of Experimental Oncology, Fondazione IRCCS Istituto Nazionale dei Tumori, Milan, Italy

**Oxford Nanopore Technologies (ONT) enables rapid, on-platform DNA methylation profiling, but validation on formalin-fixed, paraffin-embedded (FFPE) material remains limited. We evaluated ONT sequencing for molecular classification and copy-number profiling in 12 FFPE medulloblastomas. Using nanoDx, subtype calls showed substantial concordance with Illumina Infinium MethylationEPIC v2.0/DKFZ references; Sturgeon provided moderate agreement. CNV profiles derived from ONT recapitulated EPIC v2.0 alterations, including events involving MYC, TP53, and CDK6. Our results demonstrate the technical feasibility and analytical robustness of ONT in archival tissue, supporting its use as a rapid, cost-efficient complement to array-based workflows. This proof-of-concept supports broader adoption and prospective validation in clinical neuro-oncology practice.**

Oxford Nanopore Technologies (ONT) sequencing, a third-generation sequencing platform, has emerged as a powerful tool in cancer diagnostics, enabling the direct sequencing of native DNA without the need for template amplification or manipulation.^^1^^ The ability of ONT to directly assess DNA methylation provides rapid stratification of brain tumors, a critical step in determining clinical management, particularly in pediatric medulloblastoma. While classification via methylation profiling has become standard in fresh frozen tissue, long-read approaches such as ONT offer additional potential for both methylation and copy number detection.

The most common clinical specimen for solid tumors, including medulloblastoma, remains formalin-fixed, paraffin-embedded (FFPE) tissue, representing a mainstay for real-world diagnostics. However, DNA extracted from FFPE is degraded and prone to chemical modifications induced by formalin fixation, compromising the quality of high-throughput genomic assays and limiting long-read sequencing approaches. While Filser et al.^^2^^ demonstrated accurate medulloblastoma subgrouping using ONT methylation calls from fresh frozen tissue, similar validation in FFPE samples has been lacking. Here, we present a study evaluating the feasibility of ONT-based methylation profiling and CNV analysis for medulloblastoma molecular classification using DNA extracted from archival FFPE tissue.

We analyzed 12 FFPE tissue specimens from high-risk medulloblastoma patients treated within the Milan protocol with hyperfractionated accelerated radiotherapy.^3^

We employed the Reduced Representation Methylation Sequencing protocol according to ONT guidelines, preparing DNA libraries with the SQK-LSK114 kit. Each sample was sequenced using a FLO-MIN114 flow cell on a GridION device, with a minimum sequencing duration of 30 h per sample, including a single flow cell wash. Data processing was conducted using the nanoDx pipeline, which facilitates brain tumor stratification based on ONT sequencing data, utilizing the Heidelberg classifier as a reference. The nanoDx pipeline integrates alignment to the human reference genome (hg19) via minimap2 (v2.26), CNV profile generation with R/Bioconductor and the QDNAseq package (v1.2), data visualization using t-distributed Stochastic Neighbor Embedding (t-SNE), and a classification scoring system for brain tumor subtypes. A nanoDx classification score above 0.15 was considered indicative of reliable classification.^4^ All 12 samples were successfully analyzed and classified. For comparison, we ran the Sturgeon pipeline on Nanopore data (high confidence: > 0.95; confidence: 0.80–0.95; inconsistent: < 0.80).^5^ Classifications were concordant with Illumina Infinium MethylationEPIC v2.0 and the DKFZ CNS tumor classifier (https://www.molecularneuropathology.org/mnp/)^6^ (Table 1).

**Table 1.** Classification Agreement Between MethylationEPICv2 Arrays and Nanopore Sequencing

	Methylation Classifier						CNV					
	MethylationEPICv2		Nanopore				MethylationEPICv2			Nanopore		
Samples	CS	DFKZ classification	RS	nanoDX classification	Score	Sturgeon classification	CDK6 (log2 Ratio)	TP53 (log2 Ratio)	MYC (log2 Ratio)	CDK6 (log2 Ratio)	TP53 (log2 Ratio)	MYC (log2 Ratio)
OB38	0.99	Group 4	0.81	Group 4	0.99	Group 4	0.43	−0.08	−0.03	0.49	−1.00	−0.14
OB41	0.99	Group 3	0.18	Group 3	0.9	Group 3	0.04	0.22	0.36	0.70	−0.34	0.83
OB46	0.98	Group 4	0.42	Group 4	0.98	Group 4	0.40	−0.46	1.22	0.45	−1.05	1.89
OB51	0.99	Group 4	0.93	Group 4	0.99	Group 4	−0.08	−0.81	0.05	0.001	−0.78	−0.09
OB52	0.9	Group 3	0.34	Group 4	0.97	Group 3	0.36	0.10	−0.45	0.45	0.38	−0.64
OB53	0.99	SHH Activated	0.78	SHH Activated	0.99	SHH Activated	−0.05	−0.45	−0.20	0.13	−0.88	−0.15
OB55	0.93	Group 4	0.36	Group 4	0.98	Group 3	0.01	0.38	−0.36	0.30	0.55	−0.48
OB61	0.83	Group 4	0.54	Group 4	0.93	Group 4	0.76	0.6	0.46	0.84	0.50	−0.59
OS49	0.98	Group 4	0.16	Group 4	NC	NC	−0.06	ND	ND	0.53	ND	ND
OS50	0.93	Group 3	0.79	Group 3	0.99	Group 4	0.46	0.20	−0.08	0.54	0.54	−0.24
OS52	0.97	Group 3	0.71	Group 3	0.94	Group 3	0.36	0.00	−0.18	0.56	0.46	−0.71
PD89	0.99	Group 4	0.81	Group 4	0.99	Group 4	0.43	−0.15	0.20	0.65	−0.90	−0.14

Twelve FFPE medulloblastomas were profiled with Nanopore and classified by nanoDx and Sturgeon pipelines. Agreement by Fleiss’ kappa was substantial for nanoDx (κ = 0.802; *P* = .0013) and moderate for Sturgeon (κ = 0.6765; *P* = .0055). CNV data for CDK6, TP53, and MYC have been reported a log2 ratio. CS = Calibrated Score; RS = Recalibrated Score.

CNV were assessed by both the EPIC v2.0 platform and Nanopore sequencing. The data revealed a range of alterations affecting key cancer-related genes, including MYC, TP53, and CDK6 and both platforms identified regions of genomic gain and loss, confirming concordance between methods. For CDK6, most samples showed a general concordance between the two methods, with OB61 and PD89 displaying the highest amplification levels (ρ = 0.6805; CI: 0.276–0.8802; *P* = .0074). In the case of TP53, the Nanopore data indicated strong deletions in samples, such as OB46, OB51, OB53, and PD89, which were generally confirmed by the EPIC values, although with slightly reduced amplitude. Conversely, samples OB55 and OB61 showed positive values, suggesting potential gains (ρ = 0.7567; CI: 0.3874–0.9168; *P* = .0035). The MYC gene displayed high amplification in OB46 using both platforms. OB41 also showed increased MYC levels, while most other samples, including OB52, OB55, and OS52, exhibited negative values, possibly indicating losses (ρ = 0.832; CI: 0.5474 or < 0.9441; *P* = .0007). Since both platforms are designed to provide whole genome profiling, we explored consistency in CNV detecting large structural variations along genome. While Nanopore data tended to show higher signal amplitudes, the consistency with EPIC measurements reinforces its reliability in FFPE specimens (Supplementary Figure 1).

In conclusion, our findings highlight the robustness of ONT sequencing, demonstrating its ability to match established methylation profiling methods, even with FFPE-derived DNA. By confirming its potential for medulloblastoma classification using FFPE samples, we strengthen Filser et al.‘s findings, marking a significant step toward clinical translation of methylation-based tumor classification. The non-inferiority of Nanopore sequencing compared to EPIC array-based profiling reinforces its diagnostic viability, benefiting from FFPE material availability while offering a faster, more cost-effective approach per sample. Given medulloblastoma’s aggressive nature, precise molecular classification, regardless of sample type (FFPE or fresh frozen), is crucial for guiding treatment and improving patient outcomes.

## Supplementary Material

vdaf180_suppl_Supplementary_Materials_1
