# Supplementary material for: Enhancing medulloblastoma classification with nanopore sequencing of FFPE samples
Source: Neurooncol Adv. 2025 Aug 11;7(1):vdaf180. doi: 10.1093/noajnl/vdaf180 (PMC12448696; doi:10.1093/noajnl/vdaf180)
Supplement: vdaf180_suppl_Supplementary_Materials_1 [file vdaf180_suppl_supplementary_materials_1.docx]

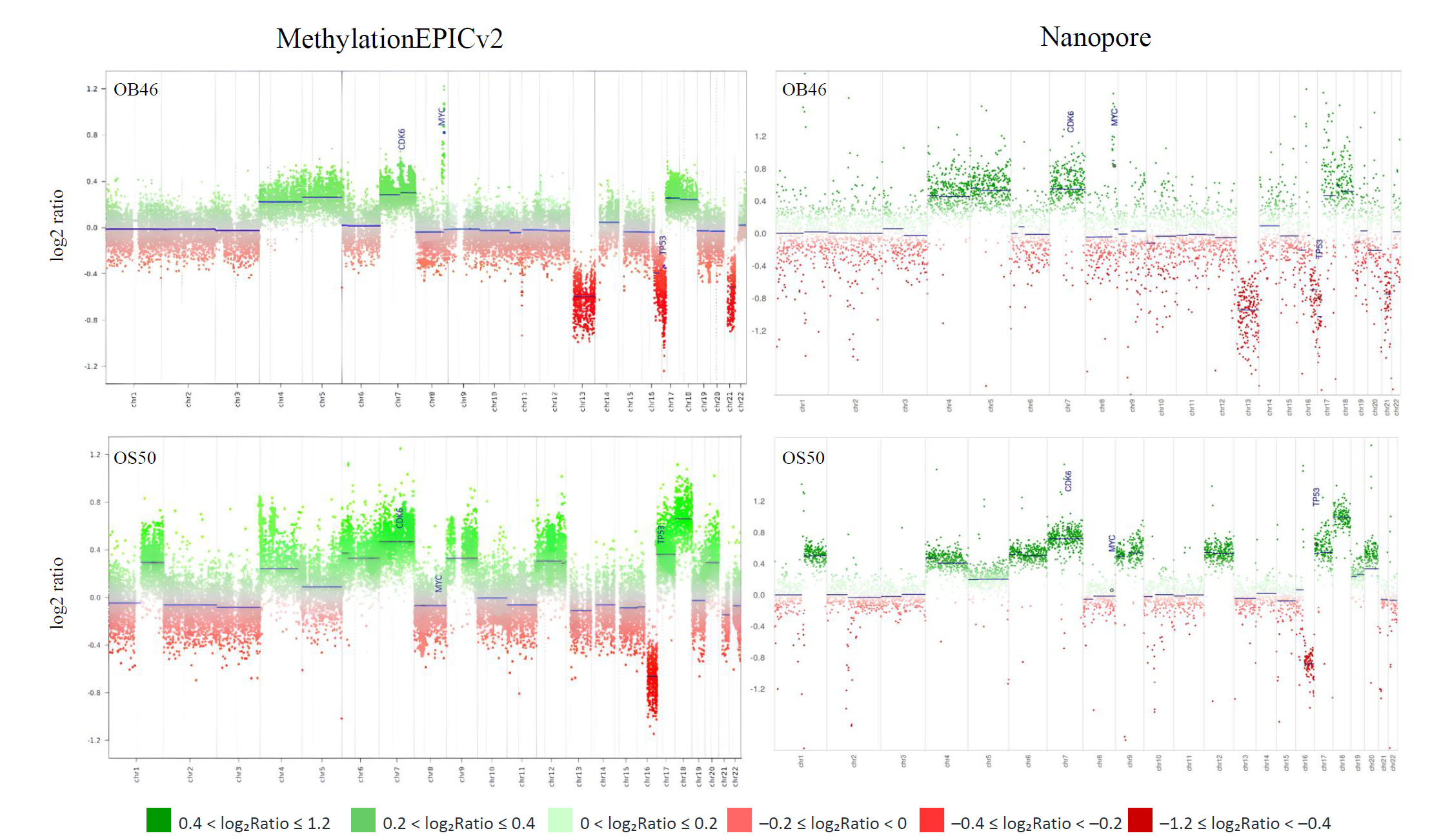


**Supplementary Figure 1:** Copy number variation (CNV) profiles for samples OB46 and OB53 obtained using EPIC v2.0 and Nanopore sequencing. Log₂ ratio values are shown for selected chromosomal regions, highlighting gains in MYC (chr8) and TP53 (chr17) in OB46, and in GLI2 (chr2) and PTCH1 (chr9) in OB53. The color scale indicates the magnitude of copy number changes.
